# Awareness and Utilization of the WHO Safe Childbirth Checklist in Facility-Based Public Health Institutions of Khordha District, Odisha: An Observational Study

**DOI:** 10.7759/cureus.108353

**Published:** 2026-05-06

**Authors:** Dharitri Swain, Latha Venkatesan

**Affiliations:** 1 Obstetrics and Gynaecological Nursing, All India Institute of Medical Sciences, Bhubaneswar, Bhubaneswar, IND; 2 College of Nursing, All India Institute of Medical Sciences, New Delhi, New Delhi, IND

**Keywords:** barriers of scc utilization, childbirth practice, quality maternal and newborn care, who safe childbirth checklist, who safe childbirth checklist awareness

## Abstract

Introduction: The WHO Safe Childbirth Checklist (SCC) is a facility-based reminder tool designed to help healthcare professionals enhance childbirth practices. We assessed the level of awareness, utilization, and barriers to utilization of the WHO SCC among obstetric health workers.

Methods: An observational cross-sectional analytical study was conducted in facility-based public health institutions of Khordha district, Odisha, India. These were primary and secondary-level public health facilities providing 24-hour maternity services to low- and middle-income women from urban and nearby rural areas. The participants comprised all obstetric care providers working in maternity units and parturient women admitted to the labor unit of public health institutions in Khordha district. These included doctors, midwives, nurses, and community health officers. The primary outcome was to assess the level of knowledge and utilization of the WHO SCC among obstetric care providers at different public health institutions. The secondary outcomes were to identify the factors associated with knowledge and utilization of the WHO SCC and perceived barriers to non-utilization of the WHO SCC.

Results: The overall mean knowledge score of obstetric care providers regarding the WHO SCC was 14.40 ± 4.91, corresponding to a mean percentage score of 48.0%, indicating suboptimal knowledge. Bivariate logistic regression analysis demonstrated that higher age (OR = 2.51; 95% CI: 1.15-5.47), professional category, i.e., midwives (OR = 2.61; 95% CI: 0.98-6.94) and nursing officers (OR = 5.00; 95% CI: 1.10-22.82), previous formal training (OR = 2.55; 95% CI: 1.05-6.16), and more clinical experience (OR = 0.37; 95% CI: 0.16-0.90) were significantly associated with higher level of knowledge.

The study further demonstrated moderate adherence (>70%) to recommended childbirth practices among providers who utilized the WHO SCC in routine labor room practice. Higher adherence was observed for key practices such as allowing a birth companion, maternal blood loss assessment after delivery, infection and eclampsia management, and discharge counseling when the SCC was used. In contrast, several essential practices, including infection and eclampsia management, delivery preparedness, breastfeeding support, and family planning counseling, showed suboptimal adherence (<70%). Healthcare providers working in hospital settings and those who had received prior training were more likely to use the SCC (p < 0.05). The predominant perceived barriers to SCC utilization included time constraints, heavy workload, staff shortages, and inadequate supervision and training.

Conclusion: Our study showed suboptimal awareness and a moderate level of adherence to recommended child birth practices. Strengthening knowledge of obstetric healthcare workers is essential to ensure consistent and effective utilization of the WHO SCC, and addressing organizational and system-level challenges may therefore be essential for improving its adherence.

## Introduction

Global concern over maternal mortality and morbidity remains substantial, particularly in low- and middle-income countries where health system constraints continue to impede safe childbirth practices. Addressing the contextual challenges of low-resource settings is essential for achieving meaningful and sustained reductions in preventable maternal deaths. In line with this, the United Nations Sustainable Development Goal 3 targets a reduction in the global maternal mortality ratio (MMR) to less than 70 per 100,000 live births by 2030, with no country exceeding twice the global average. Achieving this target requires strengthening the quality of care, improving emergency obstetric services, and ensuring equitable access to evidence-based maternal health interventions [1-4].

Identifying practical, evidence-based strategies to prevent maternal and perinatal deaths is a global priority to advance the targets of the United Nations Sustainable Development Goals (SDGs) [5]. To promote the consistent implementation of essential childbirth practices, the World Health Organization (WHO) developed the Safe Childbirth Checklist (SCC), a facility-based reminder tool aimed at supporting obstetric healthcare providers in delivering quality care during the intrapartum and immediate postpartum periods. The checklist is derived from proven, evidence-based birth practices and has been field-tested in 10 countries across Asia and Africa to improve adherence to life-saving maternal and newborn care interventions [6-8].

Childbirth is a physiological process; however, it can become unpredictable and complex, often requiring timely and sometimes advanced interventions to prevent adverse maternal and neonatal outcomes. In busy and overcrowded labor wards, healthcare providers may struggle to consistently recall and implement all essential practices in the correct sequence. To address this challenge, the WHO introduced the SCC, a simple, structured quality improvement tool that guides providers from admission through delivery and discharge [9,10].

The SCC comprises evidence-based practices derived from WHO guidelines, targeting the leading global causes of maternal and neonatal mortality. By systematically prompting critical actions at key pause points, the checklist supports adherence to life-saving interventions and promotes standardized care. Evidence suggests that consistent completion of checklist items improves the likelihood of safer birth outcomes by reinforcing fundamental clinical practices. Many studies reveal that effective implementation of the WHO SCC represents an important strategy for strengthening maternal and newborn health outcomes, particularly in resource-constrained settings of India and outside India [11-16].

Although maternal health improvement interventions have been implemented in the study area of Odisha for a considerable period, there remains a paucity of evidence regarding the current knowledge and utilization of the WHO SCC among healthcare workers. Therefore, this study was undertaken in this setting to address this gap and to assess the level of awareness, utilization, and barriers to the use of the WHO SCC across different public health institutions of Khordha district, Odisha.

The primary outcome was to assess the level of knowledge and utilization of the WHO SCC among obstetric care providers at different public health institutions. The secondary outcomes were to identify the factors associated with knowledge and utilization of the WHO SCC and perceived barriers to non-utilization of the WHO SCC.

## Materials and methods

Study design

An observational cross-sectional analytical study was conducted to assess the knowledge and utilization of the WHO SCC among obstetric care providers.

Setting

The study was conducted at primary and secondary level public maternity healthcare facilities in the Khordha district of Odisha, which is in the eastern part of India. The study was conducted for a period of 10 months (November 2024 to August 2025). The total population of the district is 22,51,673, of which approximately 11% are in the reproductive age group. Khordha is the most urbanized district of Odisha, having two subdivisions and 10 blocks [17]. The rationale for selecting this study setting was the convenience to the researcher, easy availability of the subjects, and better cooperation of the staff.

Participants

The source population comprised all obstetric care providers working in maternity units and parturient women admitted to the labor unit of public health institutions in Khordha district. The study included doctors, midwives, nurses, and community health officers. Participants comprised obstetric care providers from these professional categories who were involved in managing labor cases in the selected health institutions and who provided consent to participate in the study assessing knowledge and utilization of the WHO SCC. The samples for observing the practices of the obstetric care providers were the labor and delivery events of the parturient women admitted to the labor units of the selected health institutions.

Inclusion criteria

The total number of major public health institutions in Khordha district is 81 (10 hospitals and 71 health centers, of which 59 are newly opened primary health centers). Of these institutions, 17 health centers provide obstetric care services. The total number of obstetric care providers in these 17 health centers is 1450 (data obtained from each institution by the principal investigator from the Chief District Medical Officer (CDMO) office of the district and through a preliminary survey). All health institutions that provide obstetric care services were included in the study since the total number of health professionals working in maternity units in the study area was small. The health centers where the WHO SCC was utilized by the obstetric care providers were selected for observing the essential childbirth practice, and the events were women who were admitted to the labor unit for normal vaginal delivery. Parturient women who were referred due to the risk conditions were excluded from the study.

Outcome measures

The primary outcome was to assess the level of knowledge and utilization of the WHO SCC among obstetric care providers at different public health institutions. The secondary outcomes were to identify the factors associated with the level of knowledge and utilization of the WHO SCC and perceived barriers to non-utilization of the WHO SCC.

The WHO SCC is a structured clinical tool developed by the World Health Organization to support healthcare providers in delivering essential, evidence-based practices during childbirth. It consists of a list of evidence-based childbirth interventions organized into four key “pause points”: on admission, before pushing or cesarean section, soon after birth, and before discharge.

Knowledge regarding the WHO SCC refers to the level of understanding possessed by obstetric care providers about the purpose, components, timing (pause points), and evidence-based practices included in the WHO SCC. It was measured using a structured questionnaire.

Utilization of the WHO SCC refers to the extent to which obstetric care providers correctly and consistently apply the checklist during maternal and newborn care at all recommended pause points (on admission, before pushing/cesarean, soon after birth, and before discharge). It was measured through observation of completed checklists and expressed as a percentage or frequency of adherence to childbirth practices.

Sample size

The sample size in this cross-sectional survey was determined using a single proportion formula:

$n = Z^2 p(1 - p) / d^2$

The minimum sample size required for the study was estimated to be 196 using the above formula where *n* is the sample size, *z* is the standard normal deviate set at 1.96 (for 95% confidence level), *d* is the desired degree of precision (taken as 0.05), and *p* is the estimate of the proportion of our target population who used SCC (assumed to be 85% as obtained from pilot study).

The following finite population correction (FPC) formula was used as the total number of obstetric care providers working in the maternity units of health centers of the study area was less than 1000 (N = 200): 

$n_{\mathrm{adj}} = \frac{n}{1 + \frac{n - 1}{N}}$

Where, *n_adj_*​ = adjusted sample size (after finite population correction); *n* = initial calculated sample size; *N* = total population size.

Consequently, the corrected sample size became 100. Adjustment for a 10% rate of non-responses yielded a final sample size of 110. For practice, essential practices were done in 50% of deliveries based on the previous study conducted in Rajasthan, India [16] at 95% confidence level, and the sample size was calculated as 383 and rounded to 385.

Sampling technique

The sample was proportionally allocated to the selected health centers based on the number of obstetric care providers working in the maternity units of each institution. At each study site, enumerative sampling was used to recruit all eligible obstetric care providers within the specified time period for the assessment of knowledge. To observe labor room practices, a convenience sampling technique was employed to recruit a proportionate number of parturient women from each health facility on whom healthcare providers delivered intrapartum care in accordance with the WHO SCC.

Data collection tools

As per the objectives of the study, the demographic proforma, knowledge questionnaire, practice assessment checklist, and perceived barriers assessment tool for utilization of the WHO SCC (Appendices) were used.

The demographic proforma comprises a total of eight items of demographic specifications of the obstetric care providers. It contains one open-ended question, i.e., age in years, and seven multiple-choice questions, such as gender, educational qualification level, health institution in which the obstetric care providers work, profession, total clinical experience in maternity unit (in years), and whether they had received any training in obstetric care.

A knowledge questionnaire was used to assess the level of awareness regarding the WHO SCC and its implementation among the obstetric care providers. It consists of a total of 15 knowledge questions with two multiple-choice items under each area; hence, 30 items were included to assess their knowledge. The items were related to the meaning of the WHO SCC, components and purpose of SCC, the WHO partograph, maternal and inborn infection management, preeclampsia management, active management of the third stage of labor (AMTSL), postpartum bleeding assessment, early breastfeeding, newborn assessment for sepsis, newborn resuscitation and thermal protection, newborn feeding assessment upon discharge, discharge counseling for family planning, etc. For each correct response, one point was awarded. Therefore, the total score of the knowledge questionnaire was 30. The participants were arbitrarily categorized as having adequate knowledge and inadequate knowledge on the basis of the scores obtained. A total of 50% of the overall score, i.e., 15 points, was set as the cut-off score for further categorization. Participants scoring 15 points and above were considered to have adequate knowledge, whereas participants scoring less than 15 points were considered to have inadequate knowledge.

Essential childbirth practice assessment was measured through an observational checklist, which was used to assess actual procedures and practices being followed by the obstetric care providers in the labor units while providing intrapartum care to parturient women. It consists of 29 items related to allowing birth companions, labor preparedness, WHO partograph, maternal and inborn infection management, preeclampsia management, AMTSL, postpartum bleeding assessment, early breastfeeding, newborn assessment for sepsis, newborn resuscitation and thermal protection, newborn feeding assessment upon discharge, and discharge counseling for family planning. For each item, the observer selected YES/NO/NA based on their observation, depending on the subject’s performance on that item. Each item selected as "yes" received one point, while each "no" received 0 points. Each "NA" checked item received a score of 1, because there was no opportunity to rate that event, or that event had not occurred. Therefore, 70% of the overall score was taken as the cut-off for determining the adequate practices performed by the obstetric care providers.

The perceived barriers assessment tool was used for assessing the factors hindering the utilization of the WHO SCC, which was a five-point Likert scale containing 12 items. All the items were declarative statements in which study participants are supposed to respond in the degree of their agreement with the statements, i.e., strongly agree, agree, neutral, disagree, and strongly disagree.

Validity and reliability of the tools

The content validity of the knowledge questionnaire, practice assessment checklist, and perceived barriers assessment tool was determined through review by a panel of five experts, who were asked to comment on whether the items in the tool were relevant, appropriate, essential, clear, and acceptable. The tool, along with the blueprint of objectives and a criteria checklist, was given for validation to five experts to ensure content validity, and the items were modified accordingly. Content validity index for the knowledge questionnaire of the WHO SCC was 0.85, for the practice assessment checklist on usage of the WHO SCC was 1.0, and for the perceived barriers assessment tool was 0.75.

For assessing the reliability of the tool, the tool was administered to 10% of the total sample size. For knowledge, the knowledge questionnaire was administered to 10 obstetric care providers, and for the practice section, direct observation was done by two observers for 30 labor events. Karl Pearson’s coefficient was computed to obtain the split-half reliability of half of the test (r = 0.6403). The Spearman Brown’s Prophecy formula was used to calculate the reliability of the whole test, which came to be 0.74. This shows that the tool was reliable. For assessing the reliability of this tool, the inter-rater agreement method was used, where the tool was administered, directly observed, and rated by two separate observers on the same events. Cohen’s kappa value was calculated, which came to be k = 0.715, showing it was reliable.

Methods of data collection

The awareness level of obstetric care providers was assessed using a knowledge questionnaire, which was administered in person and via Google Forms (Google, Mountain View, CA) to those unavailable during data collection, after obtaining informed consent and providing instructions for completion. Each participant was strictly instructed to respond to the questionnaire and submit the form within 40 minutes (10 minutes for answering the demographic proforma and 30 minutes for answering the knowledge and perceived barriers assessment items). Although knowledge was assessed using a structured self-reported questionnaire, which may be prone to reporting bias, efforts were made to minimize self-reported bias by enforcing a strict time limit for questionnaire completion to reduce socially desirable or externally influenced responses.

The practices of obstetric care providers were observed using the practice assessment observational checklist by the researcher after recruiting parturient women as labor events who fulfilled the sampling criteria. A total of 385 labor events were observed directly and recorded manually while the obstetric care providers were delivering intrapartum care to those parturient women from admission to discharge in facility-based healthcare centers of Khordha district, Odisha. Direct observations and patient files were referred to collect data regarding practice in accordance with the WHO SCC. To reduce observational bias, observations were conducted by an independent third party using a standardized checklist through direct observation of 385 delivery events.

Data analysis

Data were entered and analyzed using SPSS version 21 (IBM Corp., Armonk, NY), with consistency checks performed prior to analysis. Descriptive statistics, including frequency distributions and cross-tabulations, were used to summarize quantitative variables and levels of knowledge. Bivariate and multivariable logistic regression analyses were conducted to examine associations between demographic characteristics and knowledge levels, as well as to identify predictors of SCC utilization. The strength of associations was assessed using odds ratios (OR) and adjusted odds ratios (AOR) with 95% confidence intervals. Model fitness in the multivariable logistic regression was evaluated using the Hosmer-Lemeshow goodness-of-fit test.

Ethical consideration

Ethical permission was taken from the state ethical board, and due permission was obtained from the concerned authority of the study centers before commencing data collection. Subjects were provided with a participant information sheet and were informed about the benefits of the study through the patient information sheet. Informed consent was obtained from the subjects through an informed consent sheet. Confidentiality was assured, and reassurance was given regarding the data not being used for any purpose other than research.

## Results

Demographic characteristics of the obstetric care providers

All eligible participants responded to the knowledge questionnaire and reported their utilization status of the WHO SCC, resulting in a 100% response rate. Data presented in Table 1 show the professional characteristics of the obstetric care providers. The mean age of the participants was 25.82 ± 4.1 years. A majority of the respondents were females (92, 83.64%). More than half of the participants had a degree-level qualification (63, 57.27%), followed by MBBS graduates (25, 22.73%) and diploma holders (22, 20%). Most of the participants were working in health centers (81, 73.64%), while the remaining were employed in hospitals (29, 26.36%). In terms of profession, nearly half were nursing officers (52, 47.27%), followed by doctors (25, 22.73%), midwives (20, 18.18%), and community health officers (CHOs) (13, 11.82%). Regarding clinical experience, 70 (63.64%) had more than one year of experience. A substantial proportion of participants (74, 67.27%) had received training in obstetric care.

**Table 1:** Distribution of demographic details of the obstetrics care providers (n = 110).

Variables	Frequency (f)	Percentage (%)
Age in completed years		
Mean ± SD	25.82 ± 4.1	
Gender		
Male	18	16.36%
Female	92	83.64%
Educational qualification		
Diploma	22	20%
Degree	63	57.27%
MBBS	25	22.73%
Health institution		
Hospitals	29	26.36%
Health centers	81	73.64%
Profession		
Doctor	25	22.73%
Midwife	20	18.18%
Nursing office	52	47.27%
Community health officer (CHO)	13	11.82%
Total clinical experience in maternity unit (in years)		
≤1	40	36.36%
>1	70	63.64%
Formal training received in obstetric care		
Yes	74	67.27%
No	36	32.73%

Knowledge regarding the WHO SCC

The overall mean knowledge score of the obstetric care providers on the WHO SCC was 14.40 ± 4.91, with a mean percentage of 48.0%, indicating suboptimal knowledge among participants. Adequate level of knowledge of the obstetric care providers was observed for general understanding of the WHO SCC (64, 58.18%) and knowledge related to the use of SCC during the intrapartum period (56, 51.17%), while knowledge related to the use of the SCC checklist at admission (40, 36.36%) and immediate postpartum care (46, 41.81%) was comparatively low.

Table 2 presents the proportion of healthcare professionals (n = 110) who provided correct responses to knowledge items related to the WHO SCC. All of the participants (100%) heard about the WHO SCC. Of those who heard, more than two-thirds of participants correctly knew the meaning of the WHO SCC (62, 56.36%). An equal proportion of participants (80, 72.73%) correctly identified the use of the partograph and AMTSL. Similarly, 74 (67.27%) correctly reported the overall components of the WHO SCC. Knowledge regarding early initiation of breastfeeding within one hour (71, 64.55%) and maternal postpartum bleeding assessment (69, 62.73%) was also relatively high. Moderate levels of correct responses were observed for newborn feeding assessment upon discharge (62, 56.36%), appropriate newborn resuscitation (58, 52.73%), and newborn assessment for sepsis (58, 52.73%).

**Table 2:** Correct response of healthcare professionals on the WHO SCC (n = 110). WHO: World Health Organization; SCC: Safe Childbirth Checklist.

Knowledge items	Frequency (f)	Percentage (%)
Meaning of the WHO SCC	62	56.36%
Purpose of using the SCC checklist	51	46.36%
Overall components of the WHO SCC	74	67.27%
Benefits of using the SCC checklist	51	46.36%
Preeclampsia management	41	37.27%
Maternal infection management	21	19.09%
Use of partograph	80	72.73%
Active management of the third stage of labor (AMTSL)	80	72.73%
Appropriate newborn resuscitation	58	52.73%
Maternal postpartum bleeding assessment	69	62.73%
Early breastfeeding (within one hour)	71	64.55%
Newborn assessment for sepsis	58	52.73%
Newborn feeding assessment upon discharge	62	56.36%
Postpartum counseling	37	33.64%
Counseling on family planning	35	31.82%

However, lower proportions of respondents correctly identified the purpose (51, 46.36%) and benefits (51, 46.36%) of using the SCC. Knowledge gaps were more pronounced in clinical management areas, including preeclampsia management (41, 37.27%) and maternal infection management (21, 19.09%). Additionally, only 37 (33.64%) and 35 (31.82%) of participants correctly responded to items related to postpartum counseling and family planning counseling, respectively. Overall, while respondents demonstrated adequate knowledge in selected intrapartum practices, substantial deficiencies were noted in complication management and counseling-related components of the WHO SCC.

Factors associated with the knowledge of obstetric caregivers about the WHO SCC

Table 3 presents the association between selected professional characteristics and the knowledge level of obstetric caregivers. As per the crude logistic regression analysis, age of the obstetric caregivers, professional category, clinical experience, and previous formal training showed a statistically significant association with knowledge level. Participants aged >25 years were 2.5 times more likely to have adequate knowledge compared to those aged ≤25 years (OR = 2.51; 95% CI: 1.15, 5.47). In relation to the professional category, compared with CHOs, doctors (OR = 3.50; 95% CI: 1.01-12.18), midwives (OR = 2.61; 95% CI: 0.98-6.94), and nursing officers (OR = 5.00; 95% CI: 1.10-22.82) showed higher odds of having adequate knowledge. Participants with less than one year of experience were less likely to have adequate knowledge compared to those with more than one year of experience (OR = 0.37; 95% CI: 0.16-0.90). There was no significant association established with age, professional category, or clinical experience after using multivariate logistic regression analysis.

**Table 3:** Bivariate and multivariate regression analysis of knowledge on the WHO SCC (n = 110). * Significant (p < 0.05). WHO: World Health Organization; SCC: Safe Childbirth Checklist; OR: odds ratio; AOR: adjusted odds ratio.

Variables	Overall knowledge		OR (95% CI)	AOR (95% CI)
	Inadequate, n (%) (n = 43)	Adequate, n (%) (n = 67)		
Age				
≤25	29 (67.44%)	34 (50.74%)	1	1
>25	14 (32.55%)	33 (49.25%)	2.51 (1.15-5.47)*	1.779 (483-6.556)
Gender				
Male	08 (18.60%)	10 (14.92%)	1	1
Female	35 (81.39%)	57 (85.07%)	1.303 (0.470-3.615)	0.503 (0.133-1.894)
Profession				
Doctor	15 (34.88%)	10 (14.92%)	3.500 (1.006-12.179)*	1.779 (0.644-10.422)
Midwife	06 (13.95)	14 (20.89%)	2.605 (0.979-6.936)*	0.528 (0.107-2.613)
Nursing office	19 (44.18%)	33 (49.25%)	5 (1.096-22.820)*	0.816 (0.101-6.563)
Community health officer (CHO)	03 (06.97%)	10 (14.92%)	1	1
Health institution				
Hospital	07 (16.27%)	22 (32.83%)	1	1
Health center	36 (83.72%)	45 (67.16%)	0.4 (0.15-1.03)	-
Total clinical experience				
≤1 year	10 (23.25%)	30 (44.77%)	0.37 (0.16-0.9)*	0.424 (0.102-1.755)
>1 year	33 (76.74%)	37 (55.22%)	1	1
Previous formal training				
No	34 (79.06%)	40 (59.70%)	1	1
Yes	09 (20.93%)	27 (40.29%)	2.55 (1.05-6.16)*	2.884 (1.015-8.190)*

Similarly, previous training on the WHO SCC showed a significant positive association. Participants who had received formal training were 2.55 times more likely to have adequate knowledge compared to those without prior training (OR = 2.55; 95% CI: 1.05-6.16). This association remains significant after using multivariate regression logistic analysis (AOR = 2.884; 95% CI: 1.015-8.190).

Utilization of the WHO SCC in accordance with the WHO SCC guidelines

Among 385 observed deliveries, compliance with selected components of the WHO SCC varied widely. The highest median score was observed before discharge (293, 76%), showing better adherence to essential childbirth practices in the postpartum, followed by soon after birth (192, 50%). Lower median scores were reported on admission (132, 34.28%) and during labor and delivery (101, 26.23%).

On admission, allowing a birth companion was universally practiced (385, 100%), while labor preparedness counseling was reported in 71 (81.5%); however, partograph plotting (92, 24%), hand hygiene (92, 24%), maternal infection management (171, 44.5%), and eclampsia management (50, 13.0%) were suboptimal. During labor, emergency birth companion identification was noted in 238 (61.81%), whereas the readiness of the delivery kit (92, 24%) and newborn kit (50, 13%) remained low. Within one hour of birth, AMTSL was performed in 300 (77.92%), early breastfeeding and skin-to-skin contact in 292 (76%), newborn thermal protection in 238 (61.81%), and newborn referral (if applicable) in 100%, while maternal blood loss assessment (92, 24.0%) and breastfeeding support counseling (117, 30.5%) were inconsistently practiced. Before discharge, maternal blood loss assessment was universally practiced (385, 100%), and discharge counseling was provided in 292 (76%) cases, whereas family planning counseling was documented in only 71 (18.5%) of cases (Table 4).

**Table 4:** Childbirth practices in accordance with the WHO SCC (n = 385). WHO: World Health Organization; SCC: Safe Childbirth Checklist.

Correct childbirth practices	Frequency (f)	Percentage (%)
A. On admission	n	%
Plotting of the partograph	92	24%
Appropriate maternal infection management	171	44.5%
Appropriate eclampsia management	50	13.0%
Hand hygiene	92	24%
Allowing a birth companion	385	100%
Labor preparedness counseling	71	81.5%
B. Just before pushing/during labor		
Emergency birth companion was identified	238	61.81%
Appropriate maternal infection management	109	28.31%
Appropriate eclampsia management	171	44.5%
Delivery kit and other essential supplies for mothers were ready for use	92	24%
Newborn kit and other essential supplies for babies were ready for use	50	13%
Assistance was ready to help during the delivery if necessary	92	24%
C. Soon after birth (within 1 hour)		
Active management of the third stage of labor (AMTSL) is performed	300	77.92%
Appropriate newborn thermal protection	238	61.81%
Appropriate newborn resuscitation	191	49.61%
Maternal blood loss assessment and treatment	92	24%
Appropriate maternal infection management	167	43.5%
Appropriate eclampsia management	201	52.2%
Newborn referral is done, if applicable	385	100%
Appropriate newborn infection management	167	43.5%
Newborn special care was given, if applicable	92	24%
Early breastfeeding and skin-to-skin contact are given	292	76%
Breastfeeding support counseling is done	117	30.5%
D. Before discharge		
Maternal blood loss assessment	385	100%
Appropriate maternal infection management	192	49.87%
Appropriate newborn infection management	191	49.61%
Newborn feeding assessment	117	30.5%
Family planning counseling	71	18.5%
Discharge counseling and follow-up visit	292	76%

Factors associated with utilization of the WHO SCC among obstetric caregivers

Table 5 shows predictors for utilization of the WHO SCC. Out of 110 participants, 37 reported that they had utilized the SCC in their labor room settings, and 73 never used it. The multivariable binary logistic regression model demonstrated adequate goodness of fit as indicated by the Hosmer-Lemeshow test (p > 0.05). After adjustment for potential confounders, professionals, health institutions, and previous training remained significant predictors of utilization. Obstetric caregivers working in hospitals had significantly lower odds of utilization of the WHO SCC compared to those working in health centers (AOR = 0.20; 95% CI: 0.06-0.65). Among professionals, doctors had significantly lower odds of utilization of the WHO SCC compared to nursing professionals (AOR = 0.25; 95% CI: 0.05-1.21). Participants who had received previous formal training were significantly more likely to utilize the WHO SCC in their labor room settings than those without prior training (AOR = 2.73; 95% CI: 1.03-7.21). Other variables such as age, gender, and clinical experience were not significantly associated with utilization after adjustment.

**Table 5:** Bivariate and multivariate regression analysis of utilization of the WHO SCC. * Significant (p < 0.05). WHO: World Health Organization; SCC: Safe Childbirth Checklist; OR: odds ratio; AOR: adjusted odds ratio.

Variables	Overall utilization		OR (95% CI)	AOR (95% CI)
	Not utilized (n = 73), n (%)	Utilized (n = 37), n (%)		
Age				
≤25	43 (58.9%)	20 (54.1%)	1.218 (0.549-2.704)	0.71 (0.19-2.65)
>25	30 (41.1%)	17 (45.9%)	1	
Gender				
Male	10 (13.7%)	08 (21.6%)	0.57 (0.20-1.6)	0.97 (0.28-3.35)
Female	63 (86.3%)	29 (78.4%)	1	
Profession				
Doctor	14 (19.2%)	11 (29.7%)	0.22 (0.05-0.96)*	0.25 (0.05-1.21)*
Midwife	17 (23.3%)	03 (8.1%)	0.56 (0.21-1.51)	0.25 (0.05-1.28)
Nursing office	36 (49.3%)	16 (43.2%)	1.48 (0.38-5.70)	0.46 (.06-3.23)
Community health officer (CHO)	06 (8.2%)	07 (18.9%)	1	
Health institutions				
Hospital	14 (19.2%)	15 (40.5%)	0.35 (0.15-0.84)*	0.2 (0.06-0.65)*
Health center	59 (80.8%)	22 (59.5%)	1	
Total clinical experience				
≤1 year	24 (32.9%)	16 (43.2%)	1	
>1 year	49 (67.1%)	21 (56.8%)	0.64 (0.28-1.45)	0.57 (0.15-2.14)
Previous formal training				
Yes	17 (23.3%)	17 (45.9%)	2.8 (1.204-6.513)*	2.73 (1.03-.21)*
No	56 (76.7%)	20 (54.1%)	1	

Perceived barriers that affect the utilization of the WHO SCC

Table 6 presents the perceived barriers affecting the utilization of the WHO SCC. The majority of participants identified workload (65, 89%) and time constraints (59, 87.7%) as the most significant barriers. Human resource-related factors were also perceived as hindering factors that affect the implementation of SCC in a complete form. Staff shortage affecting proper SCC use was perceived by 60 (82.2%) participants, while 59 (80.8%) stated that lack of supervision reduces SCC utilization. Furthermore, 57 (78.1%) agreed that inadequate coaching or training compromises high-risk care management.

**Table 6:** Perceived barriers that affects the utilization of the WHO SCC (n = 73). WHO: World Health Organization; SCC: Safe Childbirth Checklist.

Items	Agreement		
	Agree, n (%)	Neutral, n (%)	Disagree, n (%)
WHO SCC is time-consuming	64 (87.7%)	3 (8.1%)	6 (16.2%)
Heavy workload prevents SCC completion	65 (89.0%)	7 (18.9%)	1 (2.7%)
Time constraint to complete SCC	59 (80.8%)	4 (10.8%)	10 (27%)
Staff shortage affects proper SCC use	60 (82.2%)	8 (21.6%)	5 (13.5%)
Lack of coaching/training affects high-risk care	57 (78.1%)	10 (27.0%)	6 (16.2%)
Lack of supervision reduces SCC use	59 (80.8%)	4 (10.8%)	10 (27%)
Hospital policy needs modification	56 (76.7%)	9 (24.3%)	8 (21.6%)
Extra components in SCC add burden	55 (75.3%)	12 (32.4%)	6 (16.2%)
Difficulty in accessing instruments/equipment used in SCC	54 (74.0%)	5 (13.5%)	14 (37.8%)
Equipment non-availability hinders SCC implementation	49 (67.1%)	12 (32.4%)	12 (32.4%)
Lack of motivation reduces SCC use	47 (64.4%)	11 (29.7%)	15 (40.5%)

Organizational and structural challenges were evident, with 56 (76.7%) suggesting that hospital policies require modification to facilitate SCC use, and 55 (75.3%) reporting that additional components in the checklist increase the workload burden. Resource-related barriers were also prominent, as 54 (74.0%) reported difficulty accessing instruments or equipment required for SCC implementation, and 49 (67.1%) cited equipment non-availability as a hindrance. Lack of motivation was reported by 47 (64.4%) respondents.

Overall, the findings indicate that SCC utilization is primarily hindered by systemic factors, particularly excessive workload, staffing shortages, inadequate supervision, and logistical constraints.

## Discussion

Our study findings reported that although the SCC has been introduced as a structured tool to enhance the quality and safety of maternal and newborn care, a comprehensive understanding of its components remains inadequate among obstetric healthcare providers. There was suboptimal knowledge among obstetric healthcare providers, with an overall mean percentage of 48%. Our findings are consistent with a study conducted in parts of Ghana, which reported that 41% of healthcare workers in rural areas were aware of the WHO SCC, while 59% were not. Similar patterns are observed across many low-resource settings, where limited awareness persists due to challenges such as inadequate education and training systems, lower socioeconomic conditions, and weak regulatory and policy frameworks governing the quality of services in public health facilities [17].

This study also observed that 61% of participants had adequate knowledge of the overall aspects of the WHO SCC. In relation to different components of SCC, they had adequate knowledge on the general understanding of WHO SCC, such as meaning, components, purpose, and benefits of using SCC in labor room practice, whereas knowledge related to the use of SCC during different phases of the intrapartum period, such as at admission, during childbirth, and immediate postpartum care, was comparatively low. Our findings are supported by a study conducted in India, which reported that nearly 80% of healthcare workers had adequate knowledge of the WHO SCC [18].

In contrast, a study conducted in Somaliland reported that the majority of respondents (75%) had not heard of the WHO SCC, and nearly 95% lacked knowledge of its components, indicating a markedly poor level of awareness and understanding of the checklist [19]. In a similar study conducted in China, the obstetric medical staff scored a moderate level of knowledge on the WHO SCC (78.6%) [20].

Some professional characteristics, such as higher age of obstetric caregivers, nurses, and midwives, more clinical experience, and previous formal training, showed a statistically significant association with higher knowledge level. A similar study conducted in China, which assessed the knowledge, attitudes, and practices of obstetric healthcare staff regarding the SCC and examined its influencing factors, found that professional role, level of the hospital, and years of work experience significantly affected knowledge scores [20].

The present study reveals a moderate level of adherence to recommended practices among those who utilized the WHO SCC in routine labor room practice (>70%). Maximum adherence to several essential practices, such as allowing a birth companion, assessing maternal blood loss after delivery, managing infections and eclampsia, and providing discharge counseling, is achieved with the use of the WHO SCC. Many critical practices showed suboptimal performance, particularly those related to infection management, eclampsia management, preparedness for delivery, breastfeeding, and family counseling before discharge without the use of SCC (<70%). A similar study conducted in Sri Lanka reported average adherence to checklist practices (71.3%) [18]. Various studies have shown that non-adherence to essential birth practices affects the quality of care and neonatal consequences [21-23].

Previous studies reported significant improvement in AMTSL, postpartum bleeding assessment, newborn assessment for sepsis, newborn feeding assessment upon discharge, and discharge counseling for family planning with the WHO SCC utilization [23-26]. In contrast, some studies reported non-significant improvement of these childbirth practices [27,28]. Similar findings were reported in a study conducted in Nigeria, where only 4.8% of midwives indicated that they consistently incorporated the checklist into their routine practice. The study also recommended targeted interventions to bridge this gap, enhance awareness, and improve the utilization of the checklist during childbirth [29]. In the present study, health workers working in hospitals had significantly lower odds of utilization compared to those working in health centers, while participants who had received prior training were more likely to utilize the service.

Identifying barriers is essential to enhancing the uptake of interventions and strengthening the implementation of clinical practice strategies. Although behavioral interventions have proven effectiveness, translating evidence-based practices into routine clinical care remains a persistent challenge [29,30]. Our study investigated the perceived barriers to the utilization of the WHO SCC, which shows that time constraints, heavy workload, staff shortages, and inadequate supervision/training are the dominant perceived barriers to the WHO SCC utilization. Logistical issues such as equipment availability and individual motivation were perceived as comparatively less influential. Addressing organizational and system-level challenges may therefore be essential to improving SCC adherence. Previous studies have also identified organizational and economic factors contributing to the low utilization of the checklist. These include inadequate training, limited availability of resources and essential supplies, and systemic constraints within health facilities. Such findings underscore the need for adequate resource allocation, along with enhanced training and awareness, to ensure that all midwives effectively incorporate the checklist into routine practice [30]. Facilitating factors reported in the literature include improved interpersonal communication and teamwork promoted by the checklist. In contrast, key barriers include lack of motivation, poor acceptance of new practices, increased workload, staff shortages, and concerns regarding the user-friendliness of the SCC [18]. Therefore, implementing such programs with comprehensive training and regular monitoring is essential to ensure sustained and optimal utilization of the checklist.

Our findings underscore the need for periodic capacity-building initiatives, structured orientation programs for newly recruited staff, and reinforcement through supportive supervision. Integrating SCC training into continuing professional development sessions and simulation-based drills may improve retention and practical understanding. Strengthening knowledge across all stages of childbirth care is essential to ensure consistent and effective utilization of the WHO SCC and to optimize maternal and newborn outcomes.

Strength and limitations

This study used the WHO SCC as a quality improvement tool to assess essential childbirth practices performed by obstetric healthcare providers in primary and secondary level public maternity healthcare facilities and their perceived barriers to utilizing the WHO SCC. The study further evaluated the level of awareness, utilization pattern, and factors associated with knowledge and utilization of the WHO SCC among a large observational sample, and validated tools were used for collecting accurate data.

The limitations of the study were that it was conducted in selected primary and secondary level public maternity healthcare facilities of one district, so the results may not be generalized to the larger population or other tertiary healthcare settings. The possibility of information bias arising from self-reported responses to the knowledge questionnaire, as well as observational bias associated with the use of the WHO SCC observation checklist, may have influenced the observed associations. The childbirth practices were observed only among healthcare providers who utilized the WHO SCC. Variations in adherence patterns in settings where the checklist was not implemented were not assessed, which may limit the applicability of the findings to other healthcare contexts.

## Conclusions

This study demonstrates that knowledge and utilization of the WHO SCC among obstetric care providers remain suboptimal, with important variations across provider characteristics and facility contexts. Knowledge levels were significantly influenced by age, professional category, clinical experience, and prior formal training, while utilization was associated with professional category, type of health facility, and training exposure. Notably, nursing professionals who had received formal training showed better adherence to SCC practices, underscoring the value of capacity-building initiatives.

Persistent barriers, including high workload, time constraints, staff shortages, and inadequate supervision, continue to limit effective implementation. Addressing these challenges requires targeted strategies such as structured and ongoing training programs, strengthened supportive supervision, and improved resource allocation. Integrating these measures into routine health system practices may enhance SCC uptake, promote adherence to evidence-based practices, and ultimately improve the quality and safety of maternal and newborn outcomes.

## Appendices

Data collection tool

Instructions to the Respondents

Please try to understand the question carefully and select your answer for each item by putting a tick mark. Please complete the whole questionnaire and return it to the researcher. All the answers will be kept confidential. Thank you for your cooperation. This questionnaire consists of two components, i.e., tool A and tools B, C, and D

A. Demographic proforma

**Table 7:** Sociodemographic profile of obstetrics healthcare providers. ANM: auxiliary nurse midwife; GNM: general nurse and midwife; BSc: Bachelor of Science; MSc: Master of Science; CHC: community health center; PHC: primary health center; CHO: community health officer.

Tool A: DEMOGRAPHIC PROFORMA (Sociodemographic profile of obstetrics healthcare providers). Instructions: This tool requires information related to your personal data. Please answer each question in words whenever required and choose from alternative options when available.
Age in years:
Gender: • Male • Female
Educational qualification level: • ANM • GNM • Post-basic BSc. degree • BSc. degree • MSc. degree • MBBS
Health institution/Health center you work at: • District hospital • CHC • PHC/sub-center
Profession: • Midwife • Nursing officer • Intern doctor • Nursing intern • CHO doctor
Total experience in maternity unit (in years): • <1 year • 1-5 years • 6-10 years • >10 years
Have you received any formal training on obstetric care? • Yes • No

B. Knowledge questionnaire

**Table 8:** Knowledge questionnaire about the WHO SCC. WHO: World Health Organization; SCC: Safe Childbirth Checklist; AMTSL: active management of the third stage of labor; PPH: postpartum hemorrhage; BP: blood pressure.

Section B: KNOWLEDGE QUESTIONNAIRE ABOUT THE WHO SCC. Instruction to respondents: This section consists of multiple-choice questions, which you need to answer by choosing from the alternatives. You are allowed to choose only one option for each question. Note: Please do not access the internet or use any information manuals or mobile phone while filling the questionnaire. Your response will be kept confidential.
1. Have you ever heard about the WHO Safe Childbirth Checklist (SCC)? (a) YES (b) NO
2. Have you participated in any training or workshop about safe childbirth from any governmental or nongovernmental institutions while you were in this work? (a) YES (b) NO
3. Would you like to participate and improve your knowledge related to the WHO SCC? (a) YES (b) NO
4. What is the WHO SCC? • It is designed to help healthcare professionals ensure the delivery of essential maternal and perinatal care practices during childbirth. • It is a composite graphical record of key data (maternal and fetal) during labor entered against time on a single sheet of paper. • It is a tool that aims to support good quality, evidence-based, respectful care during labor and childbirth, irrespective of setting or level of healthcare. • It is a tool to assess the care provided during the initial intrapartum period and is used for triaging of parturient women.
5. Do you know the components of the WHO SCC? (a) YES (b) NO
6. How many sections does the WHO SCC consist of? • 5 • 4 • 6 • 8
7. At what stage of labor must the WHO SCC be implemented? • After admission till delivery. • During the second stage of delivery. • After admission till discharge of the mother. • Throughout the first stage of labor
8. At what cervical dilation (in cm) should partograph plotting be initiated? • 4 cm • 5 cm • 6 cm • 3 cm
9. Where should the first assessment finding be marked in the partograph? • On the graphical line corresponding to the time plotted and cervical dilation on the left side of the alert line. • On the alert line corresponding to the time plotted and cervical dilation. • On the graphical line corresponding to the time plotted and cervical dilation on the right side of the alert line. • On the action line corresponding to the time plotted and cervical dilation.
10. Which components are not included in the WHO SCC during the admission phase? • Component to assess fetal well-being. • Presence of birth companions. • Initial assessment, triage, and referral. • Use of any antibiotics for the management of complications (preeclampsia, eclampsia, obstructed labor, infections, preterm)
11. What will you document during delivery in the WHO SCC? • Preparation of pre-filled oxytocin and practice of AMTSL. • Assessment of danger signs of the mother and newborn. • Family planning counseling. • PPH assessment
12. What are the components included in the WHO SCC soon after birth? • Companion, pain relief, oral fluids, and posture • PPH assessment • Referral of newborn • Pain relief and oral fluids
13. What will you document in the WHO SCC before discharge of mother and baby? • PPH assessment • Assessment of danger signs of mother and newborn • Family planning counseling • All of the above
14. When will magnesium sulphate be administered to the mother? • Diastolic BP ≥110 mmHg and 3+ proteinuria • Systolic BP ≥90 mmHg, 2+ proteinuria, with severe headache, visual disturbance, epigastric pain • As prophylaxis to all mothers to prevent seizures • Systolic BP ≥110 mmHg and 2+ proteinuria
15. Which of the following is the uterotonic of choice for prophylaxis of PPH in the third stage of labor? • Syntometrine • Oxytocin • Misoprostol • Carboprost
16. Which of the following is not related to active management of the third stage of labor (AMTSL)? • 10 IU of Oxytocin • IM uterine massage reduces the duration of the third stage. Perform in only high-risk cases
17. Which of the following statements is WRONG regarding posture and position to be provided during various stages of labor? • A position of the woman’s choice should be provided during each stage of labor • You should encourage the supine position during all stages of labor • Encourage the woman to ambulate freely in the latent phase of labor • You should restrict the ambulation during the 2^nd^ stage of labor.
18. How are deviations from the normal progression of labor assessed in the WHO partograph? • By action and alert lines. • By alert column • By actions column • By fetal well-being section.
19. How are uterine contractions marked in a partograph? • With dots, lines, and shades for mild, moderate, and severe contractions. • With numbers and symbols used for denoting minutes and seconds. • Only by numbers • Only by symbols used for denoting minutes and seconds.
20. When will antihypertensive medication be administered to the mother? • If systolic BP >160 mmHg • If systolic BP >150 mmHg • If systolic BP >140 mmHg • If diastolic BP >100 mmHg
21. Antibiotics should be administered to the mother in which of the following conditions: • Mother’s temperature ≥38°C • History of foul-smelling vaginal discharge • Rupture of membranes >18 hours • All of the above conditions
22. When should the companion call for help? • Bleeding or severe abdominal pain • Severe headache or visual disturbance • Unable to urinate or urge to push • All of the above conditions
23. Which of the following preparation to be done to care for the mother immediately after birth? • Confirm single baby only (not multiple births) • Give oxytocin within 1 minute after birth • Deliver placenta 1-3 minutes after birth and massage uterus after placenta is delivered • All of the above
24. What action will be performed by the labor staff if the baby is not breathing soon after delivery EXCEPT? • Clean airway • Ventilate with bag-and-mask • Clamp and cut cord • Refer the baby immediately
25. What action will be performed on priority by the labor staff if the mother is bleeding abnormally EXCEPT? • Massage the uterus • Administer more uterotonic • Start IV fluids and keep mother warm • Administer antibiotics
26. In which of the following conditions will antibiotics be administered to the baby soon after birth? • Respiratory rate >60/min or <30/min • Chest in-drawing, grunting, or convulsions • Poor movement on stimulation • Baby’s temperature <35°C (and not rising after warming) or baby’s temperature ≥38°C • All of the above
27. What type of counseling should be done before the discharge of the mother? • Family planning counseling • Follow-up counseling • Postpartum counseling • Intrapartum counseling
28. When should the baby start breastfeeding, and skin-to-skin contact be given? • As soon as possible after delivery • When the mother feels ready to feed the baby • Within one hour of delivery • Within 30 minutes after delivery
29. What are the supplies should mothers keep ready for use before conducting deliveries?
30. What are the supplies for the baby to be kept ready for use before conducting deliveries?

C. Practice assessment checklist

**Table 9:** Practice assessment checklist on usage of the WHO SCC. WHO: World Health Organization; SCC: Safe Childbirth Checklist; AMTSL: active management of the third stage of labor.

SECTION C: PRACTICE ASSESSMENT CHECKLIST ON USAGE OF THE WHO SCC. Instruction: The observer must observe each item in the participant and choose among YES/NO/NA based on the observation. Observation is based on the events recorded in the WHO SCC and also a review of the events recorded in the case file of the parturient woman. The observer must choose any one of the options from (Incomplete/Fully complete) on the “Status of completion of SCC” based on the data obtained from observing each item in each section of the SCC.				
S. No.	Childbirth practices	YES	NO	NA
A	On admission			
	Partograph is used			
	Appropriate maternal infection management is done			
	Appropriate eclampsia management is done			
	Hand hygiene is performed			
	Birth companion present			
	Intrapartum counseling is done			
B	Just before pushing/during labor			
	Emergency birth companion is identified			
	Appropriate maternal infection management is done			
	Appropriate eclampsia management is done			
	Supplies for mothers were ready for use			
	Supplies for babies were ready for use			
	Was the assistance identified and ready to help during the delivery if necessary?			
C	Soon after birth (within 1 hour)			
	AMTSL is performed			
	Appropriate newborn thermal protection is done			
	Appropriate newborn resuscitation is done			
	Maternal blood loss assessment and treatment were done			
	Appropriate maternal infection management is done (antibiotics given)			
	Appropriate eclampsia management is done			
	Newborn referral is done, if applicable			
	Appropriate newborn infection management is done (antibiotics given)			
	Newborn special care is given			
	Early breastfeeding and skin-to-skin contact are given			
	Postpartum counseling is done			
D	Before discharge			
	Maternal blood loss assessment is done			
	Appropriate maternal infection management is done (antibiotics given)			
	Appropriate newborn infection management is done (antibiotics given)			
	Newborn feeding assessment is done			
	Family planning counseling is given			
	Discharge counseling is given			

D. Perceived barriers assessment tool for utilization of the WHO SCC

**Table 10:** The perceived barriers assessment tool for utilization of the WHO SCC. WHO: World Health Organization; SCC: Safe Childbirth Checklist.

SECTION D: THE PERCEIVED BARRIERS ASSESSMENT TOOL FOR UTILIZATION OF THE WHO SCC. Instruction to participants: This section requires respondents to put a tick mark in the boxes for each item with respect to their view.						
S. NO.	ITEMS	STRONGLY AGREE	AGREE	NEUTRAL	DISAGREE	STRONGLY DISAGREE
1.	Do you think the WHO SCC is very time-consuming?					
2.	Do you feel that due to the heavy workload, you won’t be able to complete the SCC?					
3.	Do you feel you won’t get enough time to complete the WHO SCC?					
4.	Do you feel that the extra components added in the WHO SCC make an additional time burden?					
5.	Do you think the shortage of staff affects the usage of SCC?					
6.	Do you find it difficult to get all the instruments and equipment that are used in SCC?					
7.	Have you ever felt that a lack of equipment at your facility hinders the completion of the SCC?					
8.	Do you think that it’s not possible for a single staff member to manage so many components of the SCC at a time?					
9.	Do you feel the absence of continuous coaching/training makes it difficult to manage the high-risk cases?					
10.	Do you feel that the hospital policy has to be modified to implement the WHO SCC completely?					
11.	Have you ever felt that the lack of supervision makes you reluctant to use the WHO SCC?					
12	Have you ever felt that the lack of motivation makes you reluctant to use the WHO SCC?					
